# Assessment of epidemic risk state and its change trend of public hospital in underdeveloped area in different stages

**DOI:** 10.3389/fpubh.2024.1384118

**Published:** 2024-08-06

**Authors:** Ming Yang, Jingjing Miao, Tiebing Li, Rong Jiang, Min Jiang

**Affiliations:** ^1^School of Information, Yunnan University of Finance and Economics, Kunming, China; ^2^Yunnan Key Laboratory of Service Computing, Kunming, China; ^3^Zhaotong People's Traditional Chinese Medicine Hospital, Zhaotong, China

**Keywords:** epidemic risk, risk state, public hospital, risk assessment, hospital risk

## Abstract

**Objective:**

Epidemics are sudden and rapidly spreading. Hospitals in underdeveloped areas are particularly vulnerable in case of an outbreak. This paper aims to assess the epidemic risk state and its change trend of hospitals in different epidemic stages, identify the key factors affecting hospital epidemic risk change, provide priority reference for hospital epidemic risk control, and enhance the hospital's ability to respond to sudden epidemics.

**Methods:**

Based on Grounded theory, the epidemic risk indicators that affect hospital safety are summarized. The concept of epidemic risk state and its random state space is proposed according to Markov chain theory. The impact of each indicator on the random risk state and its change is comprehensively assessed from two aspects: risk occurrence probability and risk loss. Finally, the assessment of the hospital epidemic risk state and its change at different stages is achieved.

**Results:**

The stable risk states of public hospitals in underdeveloped areas in non-epidemic stage *t*0, early epidemic stage *t*1, and outbreak stage *t*2 are ${\hat{P}}^{t0}\left(S_{n}\right)=\left\{0.142,0.546,0.220,0.093\right\}$, ${\hat{P}}^{t1}\left(S_{n}\right)=\left\{0.025,0.364,0.254,0.357\right\}$, and ${\hat{P}}^{t2}\left(S_{n}\right)=\left\{0.020,0.241,0.191,0.548\right\}$, respectively. In non-epidemic stage, the key factor in improving the hospital epidemic risk state is emergency funding. In early epidemic stage, the key factors in improving the hospital epidemic risk state are the training of medical staff in epidemic prevention skills and the management of public health. In outbreak state, the key factor in improving the hospital epidemic risk state is the training of medical staff in epidemic prevention skills and psychological awareness.

**Conclusion:**

This paper proposes the concept of epidemic risk state, providing an effective assessment method for the epidemic risk state and its change trend in public hospitals. According to the assessment, public hospitals in underdeveloped areas in different epidemic stages should adopt different risk control strategies to improve their current risk state. Blind risk control is inefficient and may even cause the epidemic risk to transition toward a more dangerous state.

## 1 Introduction

According to “Law of the People's Republic of China on the Prevention and Control of Infectious Diseases,” “Regulations on Public Health Emergencies,” and “National Public Health Emergencies Emergency Plan,” public hospitals play an important role in the emergency response system for public health emergencies and undertake the key task of preventing infectious diseases. In recent years, sudden public health emergencies, such as infectious diseases and floods have occurred frequently, posing a serious threat to public health and social stability. Public hospitals, especially in underdeveloped areas, suffer from insufficient investment in medical and health resources, imperfect management and service mechanisms, and a shortage of professional and technical personnel (1, 2). As a result, when dealing with sudden epidemics, these public hospitals are particularly vulnerable to risks and hazards due to insufficient emergency response capabilities. If a local public hospital is unable to effectively control the epidemic, it can have serious consequences for public health, medical service quality, and hospital operating funds, and can even cause the spread of regional epidemic risk.

In China, infectious diseases are considered public health emergencies, accounting for 87.5% of all public health emergencies (3). These infectious diseases usually have the characteristics of sudden occurrence and rapid spread, with great uncertainty, which can easily cause serious harm to public health and the economy. In China's 14th Five Year Plan, the “Opinions on Promoting High Quality Development of Public Hospitals” have clearly included enhancing the emergency response capabilities of public hospitals in the development requirements (4). Hospitals, as the main bearers of infectious disease prevention and control tasks in China's sudden public health emergencies, need to comprehensively analyze potential risk factors and enhance their ability to respond to sudden epidemics.

To effectively enhance the hospital's ability to respond to sudden epidemics, it is necessary to first analyze the relevant risk factors that affect hospital safety. Scholars at home and abroad have discussed the risk factors that affect hospital safety from different perspectives and pointed out the necessity of risk control. Lingyu et al. (5) emphasized the need for strengthening emergency rescue drills and medical personnel training to improve their core emergency response capabilities during infectious disease outbreaks. LeBlanc et al. (6) had recognized that medical personnel tends to make more delegation and omission errors in high-pressure work environments. Kyron et al. (7) pointed out that emergency service personnel have a higher risk of developing mental health conditions. Zhou et al. (8) also well-acknowledged that during the epidemic, medical staff are more susceptible to psychological disorders than the general population, and it is necessary to arrange work hours reasonably during the epidemic. For instance, the following studies were conducted on ensuring the quality of medical service: Brandão et al. (9) have suggested that providing psychological training to medical personnel can effectively alleviate their anxiety, consequently ensuring the safety and quality of medical service. To effectively manage the risk of an outbreak, Gail et al. proposed a competency-based approach to health human resources (HHR) planning. This approach is used to estimate the number of professionals needed during an outbreak. It was emphasized that emergency professionals need to be adequately prepared (10). Shu et al. (11) pointed out that during major health events, existing hospitals may not have the capacity to accommodate, place, and provide medical assistance to all patients. In order to address the questions outlined above, priority should be given to the establishment of emergency medical public service facilities. For example, Wuhan Fangcang shelter hospital C Hall discharged 56% of patients after the cure, while the remaining 44% were transferred to designated hospitals for further treatment after their condition stabilized. No patient died during this transition (12). This shows that reasonable planning of emergency medical public service facilities can effectively control the epidemic risk. In addition to strengthening the response of public service facilities, the public health of hospitals also has a significant impact on the control of epidemic risk. Azuma et al. (13) found that strengthening public health management in hospitals has a direct and significant effect on preventing the spread of infectious diseases. Jie et al. (14) pointed out that there are loopholes in the emergency resource management of hospitals in China, indicating that the emergency resource management capabilities of hospitals in China need to be further strengthened, especially research based on theoretical and model levels. Weiping et al. (15) emphasized that emergency funds are crucial for providing financial support for the emergency management of public emergencies. By managing and using emergency funds scientifically and reasonably, the efficiency of emergency management can be effectively improved. In addition, Campos et al. (16) suggest that temporary hospitals can be used to relieve the pressure on hospitals during emergencies and ensure the safety of medical service facilities and personnel health. Rana et al. (17), emphasized the importance of effectively regulating hospitals during an outbreak to ensure the stability of the healthcare system. Performance assessments can help improve the efficiency and job satisfaction of medical personnel while reducing turnover rates. Kabego et al. (18) pointed out that reasonable performance management will have a significant positive impact on the quality of healthcare provided by medical institutions during outbreaks. Shen et al. (19) pointed out that by establishing administrative teams, renovating infrastructure, promoting medical staff training and patient education, the spread of the epidemic can be more effectively controlled. Zhi-jun et al. (20) pointed out the construction of hospital safety management system must strengthen investment in prevention and control of hospital infection.

In summary, there are various risk influencing factors in hospitals during the epidemic period, which affect the health of personnel, the quality of medical services, and the operations safety of the entire hospital. To effectively prevent and control epidemic risk, it is necessary to conduct an assessment of epidemic risk around these related risk factors. Although there have been some studies on the assessment of epidemic risks and emergency plans (21–26), these studies have not conducted an assessment of the epidemic risk state and its change trend, ignoring the changes in epidemic risks at different stages. As a key institution for epidemic prevention and control, hospitals must accurately grasp the current epidemic risk and its change trend. By assessing the hospital epidemic risk state and its change trend, it will be possible to effectively identify key risk factors that affect hospital safety during sudden epidemics, providing priority reference for risk prevention and control in hospitals. On the other hand, a lack of understanding of potential risks can make it difficult for hospitals to allocate resources effectively in the event of an outbreak, making it challenging for hospitals to control the epidemic risk. Especially in underdeveloped areas, hospitals often lack the necessary resources and medical conditions to respond effectively to sudden epidemics. Therefore, it has become particularly important to strengthen the ability of hospitals in underdeveloped areas to respond to sudden epidemics.

This paper will focus on the characteristics of hospitals in underdeveloped areas of China, combined with the relevant influencing factors of hospital risk management during sudden outbreaks at home and abroad, to provide effective methods for assessing the epidemic risk state and its change trend of hospitals in underdeveloped areas. Through assessment, key factors affecting hospital epidemic risk in different epidemic stages will be identified, thereby providing reference for hospital risk prevention and control.

## 2 Methods

### 2.1 Epidemic risk assessment indicators

In order to assess the epidemic risk state of hospitals in underdeveloped areas, it is necessary to first sort out the risk indicators that affect the changes in hospital epidemic risk. Therefore, this paper selected medical staff from five hospitals located in underdeveloped areas of China as the interviewees, with a total of 48 interviewees. These interviewees have varying years of work experience, including managers from different hospital departments, front-line medical staff, and epidemic prevention experts involved in COVID-19 prevention and control work between 2019 and 2022. This survey involved three rounds of consultation, with 48 questionnaires issued in each round. The expert positive coefficient of respondents in all three rounds was more significant than 0.9. Apart from the interviews and surveys, this paper also summarizes and analyzes the emergent problems and potential risks that hospitals face during major health events. This analysis draws on relevant literature and media reports of significant health events from the past decade, including literature related to the COVID-19 pandemic, influenza outbreaks, and endemic disease outbreaks; this paper also paid attention to the management announcements and logs released on the official websites of different district and county hospitals during the outbreak period, and sorted out hospitals' relevant internal management information in response to the outbreak. As mentioned above, this paper collected data from multiple sources, which can analyze the risk factors that affect hospital safety from different perspectives, and cross-validate the analysis results.

Through collection, this paper has collected over 30,000 words of various types of raw data. After collecting the above raw data, this paper intends to extract a set of available hospital epidemic risk assessment indicators from the raw data based on Grounded theory. Grounded theory is a qualitative research method aimed at continuously refining core concepts based on experience and data, and ultimately classifying and summarizing relevant concepts to construct a usable theory (27). Based on Grounded theory, this paper first encodes the raw data according to different sources, using the letter Q to identify data from questionnaires, using the letter I to identify data from literature and reports, and using the letter R to identify data from hospital management materials and announcement logs. After encoding, this paper eliminated semantic duplicates through expert discussion and analysis, and sorted out 234 non-duplicate data. This paper randomly selected 170 pieces of data for constructing the epidemic risk indicator system, and the remaining 64 pieces of data were used to test the theoretical saturation of the indicators (28). Next, through semantic extraction and induction, this paper extracted 35 initial concepts with concise semantics and clear descriptions from the selected 170 pieces of data. From the perspective of hospital risk management, these initial concepts were further classified and summarized, resulting in nine categories, namely nine epidemic risk assessment indicators, as shown in Table 1.

**Table 1:** Epidemic risk assessment indicators in underdeveloped areas.

**a_j_**	**Indicators**	**The necessity of risk control**
*a*_1_	Psychological and awareness training for medical staff	When facing the epidemic, medical staff are prone to psychological disorders. If not controlled in a timely manner, it will cause serious health problems for personnel.
*a*_2_	Training on epidemic prevention skills for medical staff	To ensure the quality of medical services and the health of personnel during the epidemic, it is necessary to strengthen the training of epidemic prevention skills for medical staff.
*a*_3_	Work rights and responsibilities during the epidemic	Incomplete work arrangements or performance evaluations can easily lead to work chaos during the epidemic, and even cause psychological health problems for medical staff.
*a*_4_	Hospital emergency medical supplies	The supply of materials in underdeveloped areas is difficult, and the supply of emergency materials is insufficient, which will seriously affect the normal operation of medical services.
*a*_5_	Hospital emergency fund preparation	During the epidemic, the funding of hospitals in underdeveloped areas made it difficult to support the normal operation of the entire hospital, posing a great threat to the quality of medical services and operational management of the hospital.
*a*_6_	Hospital emergency personnel preparation	Lack of talent in underdeveloped areas and inadequate preparation of emergency personnel will seriously endanger the service quality and operational management of hospitals.
*a*_7_	Management of public service facilities in hospitals	Improper management of public service facilities will hinder the normal operation of hospitals and affect the quality of medical services.
*a*_8_	Hospital public health management	Public health is particularly important for epidemic prevention and control. Problems in public health management will seriously affect the health of hospital personnel, leading to the spread of the epidemic.
*a*_9_	Early warning supervision and management of hospitals	Lack of early warning supervision and management makes it difficult to effectively prevent and control the epidemic in the hospital, and cannot make timely adjustments to changes in the epidemic.

Finally, to verify whether the initial concepts and categories extracted are theoretically saturated, this paper extracted and summarized the remaining 64 data, and no new concepts or categories were found, indicating that the epidemic risk assessment indicators constructed in this paper are theoretically saturated.

### 2.2 Epidemic risk state and its random state space

The common risk assessment usually establishes a risk matrix from two perspectives: the probability of risk occurrence and the severity of risk losses (29, 30), thus defining the level of risk. The results obtained through the above assessment can intuitively and quantitatively describe the size of the risk. However, in practical situations, the epidemic risks faced by hospitals cannot remain unchanged due to the influence of various factors. If a fixed risk level is used to describe the risk, it is obviously insufficient to effectively describe the real hospital epidemic risk environment. Therefore, after sorting out the indicators of hospital epidemic risk through Grounded theory, in order to effectively describe the process of epidemic risk changes and achieve the assessment of risk changes, this paper defines four common discrete states *S*_*n*_, *n* = 1, 2, 3, 4 from two aspects: the probability of risk occurrence and the severity of risk losses. The meanings of each state are as follows.

(1) *S*_1_, risk disappearance state. This state is extremely safe, indicating that the risk is almost non-existent and the resulting losses can be ignored.

(2) *S*_2_, risk potential state. This state is relatively safe, indicating that the risk of the epidemic has already existed but has not caused significant damage. At this point, there is a small probability that the risk will occur, and the resulting losses will have a small impact, which can be quickly recovered.

(3) *S*_3_, risk critical state. This is a relatively dangerous state, indicating that the risk has clearly existed and the epidemic is on the verge of outbreak, causing certain losses. At this point, there is a high probability that the risk will occur and cause certain risk losses, which will take a long time to recover.

(4) *S*_4_, risk outbreak state. This state is very dangerous, indicating that the epidemic risk has fully erupted and caused extremely serious losses, and the damage caused is difficult to recover.

**Figure 1:**
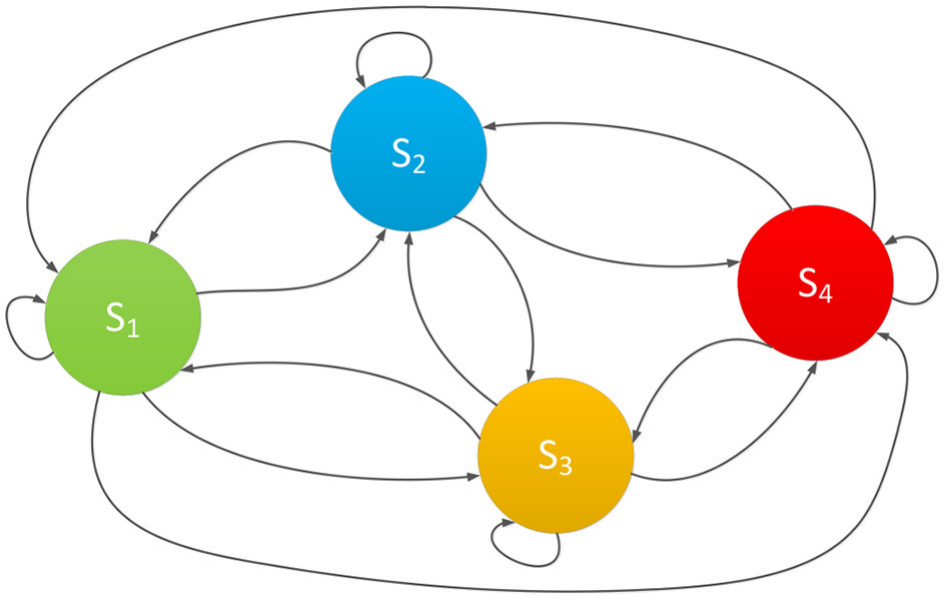
The random state space of epidemic risk.

These above four states cover the entire process of changes in epidemic risk. The probability that the epidemic risk belongs to state *S*_*n*_ is represented by *P*(*S*_*n*_), and the epidemic risk state at time *t* can be expressed as $P^{t}\left(S_{n}\right)=\left\{P^{t}\left(S_{1}\right),P^{t}\left(S_{2}\right),P^{t}\left(S_{3}\right),P^{t}\left(S_{4}\right)\right\}$,$\overset{2}{\underset{i=0}{\sum }}P^{t}\left(S_{n}\right)=1$. It is known that in practical situations, the epidemic risk is always constantly changing due to the impacts of various risk indicators. It will randomly transition between the 4 states mentioned above, as shown in Figure 1.

In Figure 1, the four risk states are mutually reachable, together forming the random state space of epidemic risk (31). Under the influence of various indicators, the actual epidemic risk in the actual situation will be randomly changing, and the entire transformation process is a random change process. Therefore, in order to effectively measure the changes in the epidemic risk state, this paper intends to adopt the Markov chain prediction method to assess the epidemic risk state and its change. Markov chain have a mathematical definition and are classic theories in probability theory and mathematical statistics. It can effectively describe the random state of things and is suitable for predicting changes in the random state of things (32).

In order to effectively assess the random transition process of the epidemic risk state under the comprehensive influence of various indicators, this paper intends to use matrix to describe the random transition process of the epidemic based on Markov chain theory. As shown below.


$$\begin{array}{l} S_{nm}=\left[\begin{array}{c} \begin{array}{ccc} S_{1\to 1} & S_{1\to 2} & \begin{array}{cc} S_{1\to 3} & S_{1\to 4} \end{array} \end{array}\\ \begin{array}{cc} S_{2\to 1}\&S_{2\to 2} & \begin{array}{cc} S_{2\to 3} & S_{2\to 4} \end{array} \end{array}\\ \begin{array}{cc} \begin{array}{c} S_{3\to 1}\\ S_{4\to 1} \end{array}\&\begin{array}{c} S_{3\to 2}\\ S_{4\to 2} \end{array} & \begin{array}{cc} \begin{array}{c} S_{3\to 3}\\ S_{4\to 3} \end{array} & \begin{array}{c} S_{3\to 4}\\ S_{4\to 4} \end{array} \end{array} \end{array} \end{array}\right] \end{array}$$

The element *S*_*n*→*m*_ in the matrix represents the process of the epidemic risk transition from state *S*_*n*_ to *S*_*m*_. The meanings of each element *S*_*n*→*m*_ are shown in Table 2.

**Table 2:** The meaning of each state transition process.

	**S_1_**	**S_2_**	**S_3_**	**S_4_**
*S*_1_	Maintain the risk disappearance state unchanged.	The risk gradually increases, transitioning from risk disappearance state to risk potential state.	Suddenly facing significant epidemic risks. Directly transitioning from risk disappearance state to risk critical state.	Epidemic risk outbreak. Directly transitioning from risk disappearance state to risk outbreak state.
*S*_2_	Risk is controlled and gradually decreases, transitioning from risk potential state to risk disappearance state.	Maintain the risk potential state unchanged.	The risk gradually increases, transitioning from risk potential state to risk critical state.	Epidemic risk outbreak. Directly transitioning from risk potential state to risk outbreak state.
*S*_3_	Risk is well-controlled, transitioning from risk critical state to risk disappearance state.	Risk is controlled and gradually decreases, transitioning from risk critical state to risk potential state.	Maintain the risk critical state unchanged.	The risk gradually increases, transitioning from risk critical state to risk outbreak state.
*S*_4_	Risk is fully controlled, transitioning from risk outbreak state to risk disappearance state.	Risk is well-controlled, transitioning from risk outbreak state to risk potential state.	Risk is controlled and gradually decreases, transitioning from risk outbreak state to risk critical state.	Maintain the risk outbreak state unchanged.

### 2.3 Assessment of the impact of various indicators on hospital risk state

After defining the epidemic risk state and its random state space, this paper will comprehensively assess the impact of each indicator on the epidemic risk state from two aspects: the probability of risk occurrence and risk loss. The level definition of the risk occurrence probability *P*(*a*_*j*_) and risk loss *L*(*a*_*j*_) for each indicator *a*_*j*_ are shown in Table 3.

**Table 3:** The level definition of the risk occurrence probability *P*(*a*_*j*_) and risk loss *L*(*a*_*j*_).

**Lev**	**Meaning**	**Lev**	**Meaning**
*P*(*a*_*j*_) = 1	Risk is almost impossible to occur	*L*(*a*_*j*_) = 1	The risk loss can be negligible, and the damage caused can be automatically restored
*P*(*a*_*j*_) = 2	Risk is less likely to occur	*L*(*a*_*j*_) = 2	The risk loss is relatively small, and the damage caused can be quickly restored
*P*(*a*_*j*_) = 3	The probability of risk occurrence is average	*L*(*a*_*j*_) = 3	The risk loss is average, and the damage caused requires some time to recover
*P*(*a*_*j*_) = 4	Risk is more likely to occur	*L*(*a*_*j*_) = 4	The risk loss is significant, and the damage caused requires a long time to recover
*P*(*a*_*j*_) = 5	Risk is almost inevitable	*L*(*a*_*j*_) = 5	The risk loss is extremely serious, and the damage caused is almost impossible to fully recover

Next, based on the definition of the four states of epidemic risk in this paper, the probability level *P*(*a*_*j*_) and risk loss level *L*(*a*_*j*_) of the risk occurrence are substituted into the risk matrix (33), which can intuitively and effectively distinguish different epidemic states, as shown in Table 4.

**Table 4:** Classification of epidemic risk state based on risk matrix.

	***L* = 1**	***L* = 2**	***L* = 3**	***L* = 4**	***L* = 5**
*P* = 5	5 (*S*_2_)	10 (*S*_3_)	15 (*S*_4_)	20 (*S*_4_)	25 (*S*_4_)
*P* = 4	4 (*S*_2_)	8 (*S*_2_)	12 (*S*_3_)	16 (*S*_4_)	20 (*S*_4_)
*P* = 3	3 (*S*_1_)	6 (*S*_2_)	9 (*S*_2_)	12 (*S*_3_)	15 (*S*_4_)
*P* = 2	2 (*S*_1_)	4 (*S*_1_)	6 (*S*_2_)	8 (*S*_2_)	10 (*S*_3_)
*P* = 1	1 (*S*_1_)	2 (*S*_1_)	3 (*S*_1_)	4 (*S*_2_)	5 (*S*_2_)

**Figure 2:**
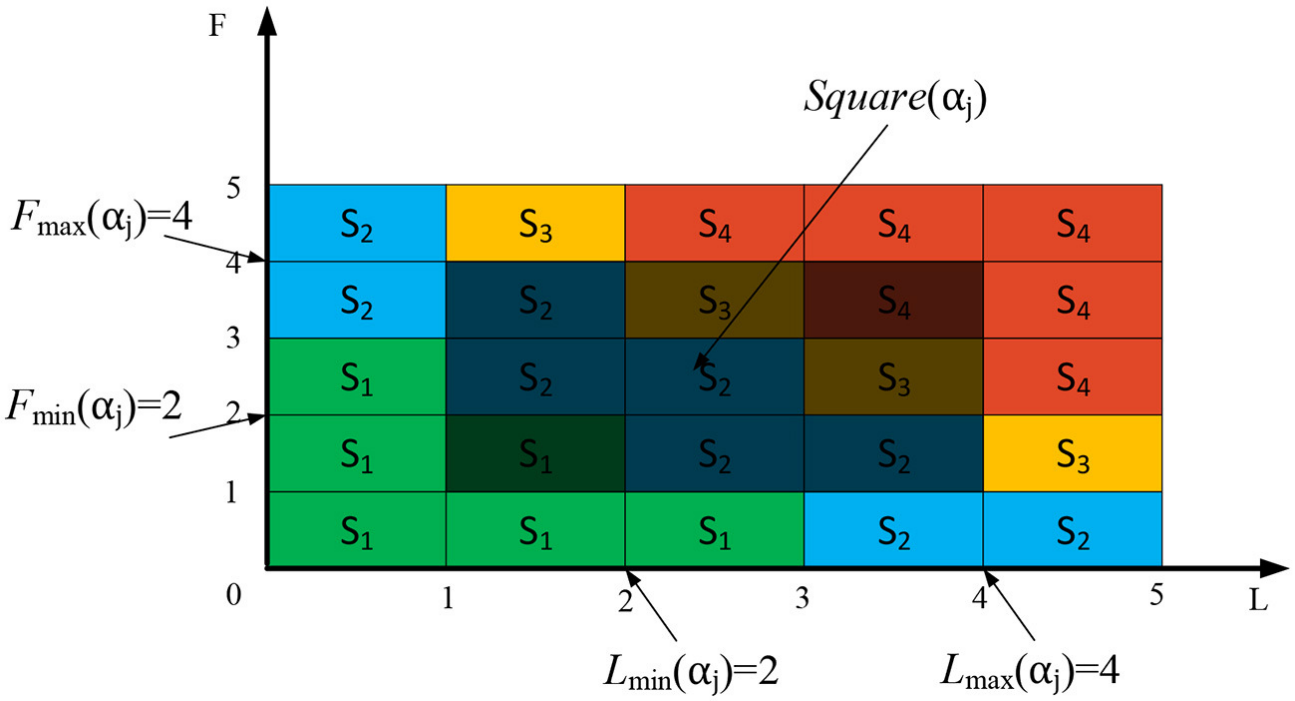
Assessment of the impact of various indicators on the epidemic risk state.

As shown in Table 4, the higher the *P*(*a*_*j*_) and *L*(*a*_*j*_), the higher the probability of risk occurrence and the greater the loss caused by the indicator. This indicator *a*_*j*_ will lead the hospital risk state to transition toward a more dangerous risk state. As mentioned above, it is only necessary to assess the probability level *P*(*a*_*j*_) and risk loss level *L*(*a*_*j*_) of each indicator, the comprehensive impact of each indicator *a*_*j*_ on the hospital's epidemic risk state can be determined. However, during the assessment process, due to the lack of expert experience, it is difficult to provide accurate judgments on *P*(*a*_*j*_) and *L*(*a*_*j*_) of a certain indicator, which can easily lead to inaccurate assessment results. To reduce the difficulty of expert assessment and ensure the accuracy of the assessment results, this paper proposes an epidemic risk state assessment method based on a risk matrix. As shown in Figure 2, experts only need to provide the value range of *P*(*a*_*j*_) and the value range of *L*(*a*_*j*_), thus the impact of indicator *a*_*j*_ on risk state can be assessed.

In Figure 2, *P*_max_(*a*_*j*_) represents the maximum level of risk occurrence probability for *a*_*j*_, while *P*_min_(*a*_*j*_) represents the minimum level of risk occurrence probability for *a*_*j*_.*L*_max_(*a*_*j*_) represents the maximum level of risk loss for *a*_*j*_, while *L*_min_(*a*_*j*_) represents the minimum level of risk loss for *a*_*j*_. The following concepts can be derived from Figure 2.

(1) *Square*(*a*_*j*_) is a rectangular area composed of four points, *P*_min_(*a*_*j*_), *P*_max_(*a*_*j*_), *L*_min_(*a*_*j*_), and *L*_max_(*a*_*j*_).
(2) *Square*(*S*_*n*_) represents the area occupied by risk state *S*_*n*_ in the risk matrix.
(3) The intersection of *Square*(*a*_*j*_) and *Square*(*S*_*n*_) indicates the possibility that *a*_*j*_ belongs to *S*_*n*_. If *Square*(*a*_*j*_)∩*Square*(*S*_*n*_) = 0, then the probability of *a*_*j*_ belonging to *S*_*n*_ is 0.

According to the above concepts, dividing the intersection of *Square*(*a*_*j*_) and *Square*(*S*_*n*_) by *Square*(*a*_*j*_) can calculate the probability that *a*_*j*_ belongs to the risk state *S*_*n*_, as shown in Formula (1).


(1) $$\begin{array}{l} \mu_{S_{n}}\left(a_{j}\right)=\frac{\text{Square}\left({\text{a}}_{\text{j}}\right)\cap \text{Square}\left({\text{S}}_{\text{n}}\right)}{\text{Square}\left({\text{a}}_{\text{j}}\right)} \end{array}$$

In Formula (1), $\mu_{S_{n}}(a_{j})$ represents the probability that *a*_*j*_ belongs to the risk state *S*_*n*_. The larger its value, the greater the probability that *a*_*j*_ belongs to *S*_*n*_, indicating a higher probability of the epidemic risk state transitioning to *S*_*n*_ under the influence of *a*_*j*_.

As mentioned above, through the method proposed in this paper, experts do not need to provide an exact numerical value for the frequency level or loss severity level of each risk indicator *a*_*j*_ in the assessment process. Experts only need to provide the value range [*P*_min_(*a*_*j*_), *P*_max_(*a*_*j*_)] and [*L*_min_(*a*_*j*_), *L*_max_(*a*_*j*_)] for each indicator according to the definition in Table 3, thus can calculate the value of $\mu_{S_{n}}(a_{j})$ according to Formula (1). $\mu_{S_{n}}(a_{j})$ effectively reflects the impact of indicator *a*_*j*_ on the risk state of the epidemic. This method reduces the difficulty of expert assessment and effectively achieves the assessment of the impact of each indicator on the epidemic risk state.

### 2.4 Assessment of the change trend of epidemic risk state

It is known that $\mu_{S_{n}}(a_{j})$ represents the probability that indicator *a*_*j*_ belongs to the risk state *S*_*n*_. When $\mu_{S_{n}}(a_{j})$ > 0, it indicates that under the influence of *a*_*j*_, the epidemic risk state of the hospital may transition to *S*_*n*_. Therefore, this paper intends to comprehensively assess the impact of *a*_*j*_ on the epidemic risk state of hospitals, and further assess the changes in the overall epidemic risk state of hospitals. As shown in Formula (2).


(2) $$\begin{array}{l} P^{{\;}^{\prime }}\left(S_{n\to m}\right)=\overset{total}{\underset{j=1}{\sum }}\mu_{S_{n}}\left(a_{j}\right),\forall \mu_{S_{n}}\left(a_{j}\right)>0 \end{array}$$

In Formula (2), $P^{{\;}^{\prime }}\left(S_{n\to m}\right)$ represents the total probability of the epidemic risk state transitioning from *S*_*n*_ to *S*_*m*_ due to the comprehensive impact of indicator *a*_*j*_. *total* represents the total number of risk indicators *a*_*j*_. For example, when *n* = 1, the values of $P^{{\;}^{\prime }}\left(S_{1\to 1}\right),\;P^{{\;}^{\prime }}\left(S_{1\to 2}\right),P^{{\;}^{\prime }}\left(S_{1\to 3}\right)$, and $P^{{\;}^{\prime }}\left(S_{1\to 4}\right)$ can be calculated sequentially according to Formula (2). Finally, according to Formula (2, the following matrix can be calculated.


$$\begin{array}{l} \left|\begin{array}{ccc} P^{{\;}^{\prime }}\left(S_{1\to 1}\right) & P^{{\;}^{\prime }}\left(S_{1\to 2}\right) & \begin{array}{cc} P^{{\;}^{\prime }}\left(S_{1\to 3}\right) & P^{{\;}^{\prime }}\left(S_{1\to 4}\right) \end{array}\\ P^{{\;}^{\prime }}\left(S_{2\to 1}\right) & P^{{\;}^{\prime }}\left(S_{2\to 2}\right) & \begin{array}{cc} P^{{\;}^{\prime }}\left(S_{2\to 3}\right) & P^{{\;}^{\prime }}\left(S_{2\to 4}\right) \end{array}\\ \begin{array}{c} P^{{\;}^{\prime }}\left(S_{3\to 1}\right)\\ P^{{\;}^{\prime }}\left(S_{4\to 1}\right) \end{array} & \begin{array}{c} P^{{\;}^{\prime }}\left(S_{3\to 2}\right)\\ P^{{\;}^{\prime }}\left(S_{4\to 2}\right) \end{array} & \begin{array}{cc} \begin{array}{c} P^{{\;}^{\prime }}\left(S_{3\to 3}\right)\\ P^{{\;}^{\prime }}\left(S_{4\to 3}\right) \end{array} & \begin{array}{c} P^{{\;}^{\prime }}\left(S_{3\to 4}\right)\\ P^{{\;}^{\prime }}\left(S_{4\to 4}\right) \end{array} \end{array} \end{array}\right| \end{array}$$

This matrix corresponds to the random state space of epidemic risk proposed in Section 2.2 of this paper, which describes the random transition probability of the epidemic risk state under the influence of various indicators. Next, normalize the elements in each row of the above matrix, and the Markov chain transition matrix of the epidemic state can be obtained. The matrix is shown below.


$$\begin{array}{l} STM=\left|\begin{array}{ccc} P\left(S_{1\to 1}\right) & P\left(S_{1\to 2}\right) & \begin{array}{cc} P\left(S_{1\to 3}\right) & P\left(S_{1\to 4}\right) \end{array}\\ P\left(S_{2\to 1}\right) & P\left(S_{2\to 2}\right) & \begin{array}{cc} P\left(S_{2\to 3}\right) & P\left(S_{2\to 4}\right) \end{array}\\ \begin{array}{c} P\left(S_{3\to 1}\right)\\ P\left(S_{4\to 1}\right) \end{array} & \begin{array}{c} P\left(S_{3\to 2}\right)\\ P\left(S_{4\to 2}\right) \end{array} & \begin{array}{cc} \begin{array}{c} P\left(S_{3\to 3}\right)\\ P\left(S_{4\to 3}\right) \end{array} & \begin{array}{c} P\left(S_{3\to 4}\right)\\ P\left(S_{4\to 4}\right) \end{array} \end{array} \end{array}\right| \end{array}$$

*STM* is the state transition matrix of epidemic risk. According to Markov chain theory, once the state transition matrix of epidemic risk is established, the stable state of things in long-term random changes can be predicted (34). The calculation formula is as follows.


(3) $$\begin{array}{l} P^{t+k}\left(S_{n}\right)=P^{t}\left(S_{n}\right)STM^{k} \end{array}$$

In Formula 3, $P^{t}\left(S_{n}\right)$ represents the epidemic risk state at time *t*, and $P^{t+k}\left(S_{n}\right)$ represents the epidemic risk state at time *t*+*k*. *k* is the number of state transitions, and *k* > 0. According to Markov chain theory, when the value of *k* is sufficiently large or approaches infinity, $P^{t+\infty }\left(S_{n}\right)$ will tend to stabilize and no longer change. The risk state at this time is called the stable state of epidemic risk, denoted as $\hat{P}\left(S_{n}\right)$. $\hat{P}\left(S_{n}\right)$ is the state where the epidemic risk state is most likely to transition. It represents the change trend of the epidemic risk state under the comprehensive influence of various indicators in a long-term risk environment. Therefore, by calculating the $\hat{P}\left(S_{n}\right)$, it will be possible to grasp the future risk trends of hospitals and effectively predict the future hospital epidemic risk state.

At this point, this paper proposes the concept of epidemic risk state, and combines risk matrix and Markov chain to propose an assessment method for epidemic risk state and its change trend. In subsequent chapters, this paper will use this method to assess the risk state and its change trend of hospitals in underdeveloped areas. The above methods were carried out in accordance with relevant guidelines and regulations, and did not involve experiments on humans and/or the use of human tissue samples.

## 3 Results

Hospitals in underdeveloped areas of China, due to their inadequate conditions, are unable to effectively respond to sudden epidemics, which can easily lead to the spread of the epidemic from remote areas and cause extremely serious local epidemic disasters. In order to effectively assess the epidemic risk state and its change trend of such hospitals, this paper selected 10 personnel with hospital risk control experience as assessment experts. These 10 experts are five hospital managers and five epidemic prevention experts from research institutes among the 48 respondents mentioned above. Based on the risk indicators proposed in this paper, through research and expert discussions on five county-level hospitals in underdeveloped areas of southwestern China, the risk indicator characteristics of public hospitals in these underdeveloped areas were summarized, as shown in Table 5.

**Table 5:** Characterization of various risk indicators for hospitals in underdeveloped areas.

**a_j_**	**Characterization**
*a*_1_	Conducted more than one training session on the psychology and awareness of medical staff about epidemic prevention.
*a*_2_	No or only one training on epidemic prevention skills for medical staff has been conducted.
*a*_3_	No specific work rights, responsibilities, or performance assessment system has been established during the epidemic period.
*a*_4_	Emergency supplies in hospitals are extremely scarce. Material supply is quite difficult.
*a*_5_	The hospital's emergency funds are relatively scarce. The turnover of funds is relatively difficult.
*a*_6_	The emergency personnel in the hospital are extremely insufficient and do not have professional epidemic prevention personnel.
*a*_7_	Public service facilities are relatively complete and can only support public services during non-epidemic periods.
*a*_8_	The public health management in hospitals is average, and there are no obvious health management issues.
*a*_9_	The hospital does not have a daily warning and supervision system in place.

Firstly, this paper selected 10 personnel with hospital risk management experience as assessment experts. In order to identify the key factors affecting the hospital epidemic risk state change in the region, this paper intends to assess the risk state of hospitals in different epidemic stages separately. These three stages are the non-epidemic stage *t*0, the early epidemic stage *t*1, and the epidemic outbreak stage *t*2.

According to the assessment method proposed in Section 2.3, 10 experts assessed the risk occurrence probability level and risk loss level of each indicator in the above three stages, and the results are shown in Table 6.

**Table 6:** The assessment results of *P*(*a*_*j*_) and *L*(*a*_*j*_) in different epidemic stages.

	****t**0**				****t**1**				****t**2**			
	**P**_**min**_ **(a**_**j**_ **)**	**P**_**max**_ **(a**_**j**_ **)**	**L**_**min**_ **(a**_**j**_ **)**	**L**_**max**_ **(a**_**j**_ **)**	**P**_**min**_ **(a**_**j**_ **)**	**P**_**max**_ **(a**_**j**_ **)**	**L**_**min**_ **(a**_**j**_ **)**	**L**_**max**_ **(a**_**j**_ **)**	**P**_**min**_ **(a**_**j**_ **)**	**P**_**max**_ **(a**_**j**_ **)**	**L**_**min**_ **(a**_**j**_ **)**	**L**_**max**_ **(a**_**j**_ **)**
*a*_1_	1	3	4	5	3	4	4	5	3	4	4	5
*a*_2_	1	3	2	4	3	4	2	4	4	5	2	4
*a*_3_	2	3	1	3	3	4	1	3	3	4	1	3
*a*_4_	2	4	3	4	4	5	3	4	5	5	3	4
*a*_5_	2	4	4	4	4	5	4	4	5	5	4	4
*a*_6_	2	4	3	4	4	5	3	4	5	5	3	4
*a*_7_	1	3	3	4	4	5	3	4	5	5	3	4
*a*_8_	2	3	2	4	3	4	2	4	4	4	2	4
*a*_9_	2	4	2	3	3	4	2	3	3	4	2	3

The data in Table 6 are the value range given by experts for the risk occurrence probability level and risk loss level of various indicators. Next, substitute Table 6 data into Formula 1, the $\mu_{S_{n}}(a_{j})$ of each indicator can be calculated, as shown in Table 7.

**Table 7:** Assessment results of each indicator's impact on the epidemic risk state in different epidemic stages.

	***t***0				***t***1				***t***2			
	μ_*S*_1__(*a*_*j*_)	μ_*S*_2__(*a*_*j*_)	μ_*S*_3__(*a*_*j*_)	μ_*S*_4__(*a*_*j*_)	μ_*S*_1__(*a*_*j*_)	μ_*S*_2__(*a*_*j*_)	μ_*S*_3__(*a*_*j*_)	μ_*S*_4__(*a*_*j*_)	μ_*S*_1__(*a*_*j*_)	μ_*S*_2__(*a*_*j*_)	μ_*S*_3__(*a*_*j*_)	μ_*S*_4__(*a*_*j*_)
*a*_1_	0.0000	0.5000	0.3333	0.1667	0.0000	0.0000	0.2500	0.7500	0.0000	0.0000	0.2500	0.7500
*a*_2_	0.3333	0.5556	0.1111	0.0000	0.0000	0.5000	0.3333	0.1667	0.0000	0.1667	0.3333	0.5000
*a*_3_	0.5000	0.5000	0.0000	0.0000	0.1667	0.6667	0.1667	0.0000	0.1667	0.6667	0.1667	0.0000
*a*_4_	0.0000	0.5000	0.3333	0.1667	0.0000	0.0000	0.2500	0.7500	0.0000	0.0000	0.0000	1.0000
*a*_5_	0.0000	0.3333	0.3333	0.3333	0.0000	0.0000	0.0000	1.0000	0.0000	0.0000	0.0000	1.0000
*a*_6_	0.0000	0.5000	0.3333	0.1667	0.0000	0.0000	0.2500	0.7500	0.0000	0.0000	0.0000	1.0000
*a*_7_	0.1667	0.6667	0.1667	0.0000	0.0000	0.0000	0.2500	0.7500	0.0000	0.0000	0.0000	1.0000
*a*_8_	0.1667	0.6667	0.1667	0.0000	0.0000	0.5000	0.3333	0.1667	0.0000	0.3333	0.3333	0.3333
*a*_9_	0.1667	0.6667	0.1667	0.0000	0.0000	0.7500	0.2500	0.0000	0.0000	0.7500	0.2500	0.0000

In Table 7, $\mu_{S_{n}}(a_{j})$ represents the probability of each indicator belonging to different epidemic risk states during different epidemic stages. For example, in the *t*0 stage, when μ_*S*_3__(*a*_1_)>0, it indicates that under the influence of *a*_1_, the overall epidemic risk state may transition to *S*_3_. Next, by substituting Table 7 data into Formula 2 and normalizing it, the state transition matrices of hospital epidemic risk in three different stages can be obtained. The results are shown below.


$$\begin{array}{l} STM^{t0}=\left|\begin{array}{ccc} 0.2667 & 0.6111 & \begin{array}{cc} 0.1222 & 0.0000 \end{array}\\ 0.1482 & 0.5432 & \begin{array}{cc} 0.2160 & 0.0926 \end{array}\\ \begin{array}{c} 0.1042\\ 0.0000 \end{array} & \begin{array}{c} 0.5486\\ 0.4583 \end{array} & \begin{array}{cc} \begin{array}{c} 0.2430\\ 0.3333 \end{array} & \begin{array}{c} 0.1042\\ 0.2084 \end{array} \end{array} \end{array}\right|\\ STM^{t1}=\left|\begin{array}{ccc} 0.1667 & 0.6666 & \begin{array}{cc} 0.1667 & 0.0000 \end{array}\\ 0.0417 & 0.6042 & \begin{array}{cc} 0.2708 & 0.0833 \end{array}\\ \begin{array}{c} 0.0208\\ 0.0000 \end{array} & \begin{array}{c} 0.3021\\ 0.1429 \end{array} & \begin{array}{cc} \begin{array}{c} 0.2604\\ 0.2381 \end{array} & \begin{array}{c} 0.4167\\ 0.6191 \end{array} \end{array} \end{array}\right|\\ STM^{t2}=\left|\begin{array}{ccc} 0.1667 & 0.6666 & \begin{array}{cc} 0.1667 & 0.0000 \end{array}\\ 0.0417 & 0.4792 & \begin{array}{cc} 0.2708 & 0.2083 \end{array}\\ \begin{array}{c} 0.0333\\ 0.0000 \end{array} & \begin{array}{c} 0.3833\\ 0.0714 \end{array} & \begin{array}{cc} \begin{array}{c} 0.2667\\ 0.1309 \end{array} & \begin{array}{c} 0.3167\\ 0.7976 \end{array} \end{array} \end{array}\right| \end{array}$$

**Figure 3:**
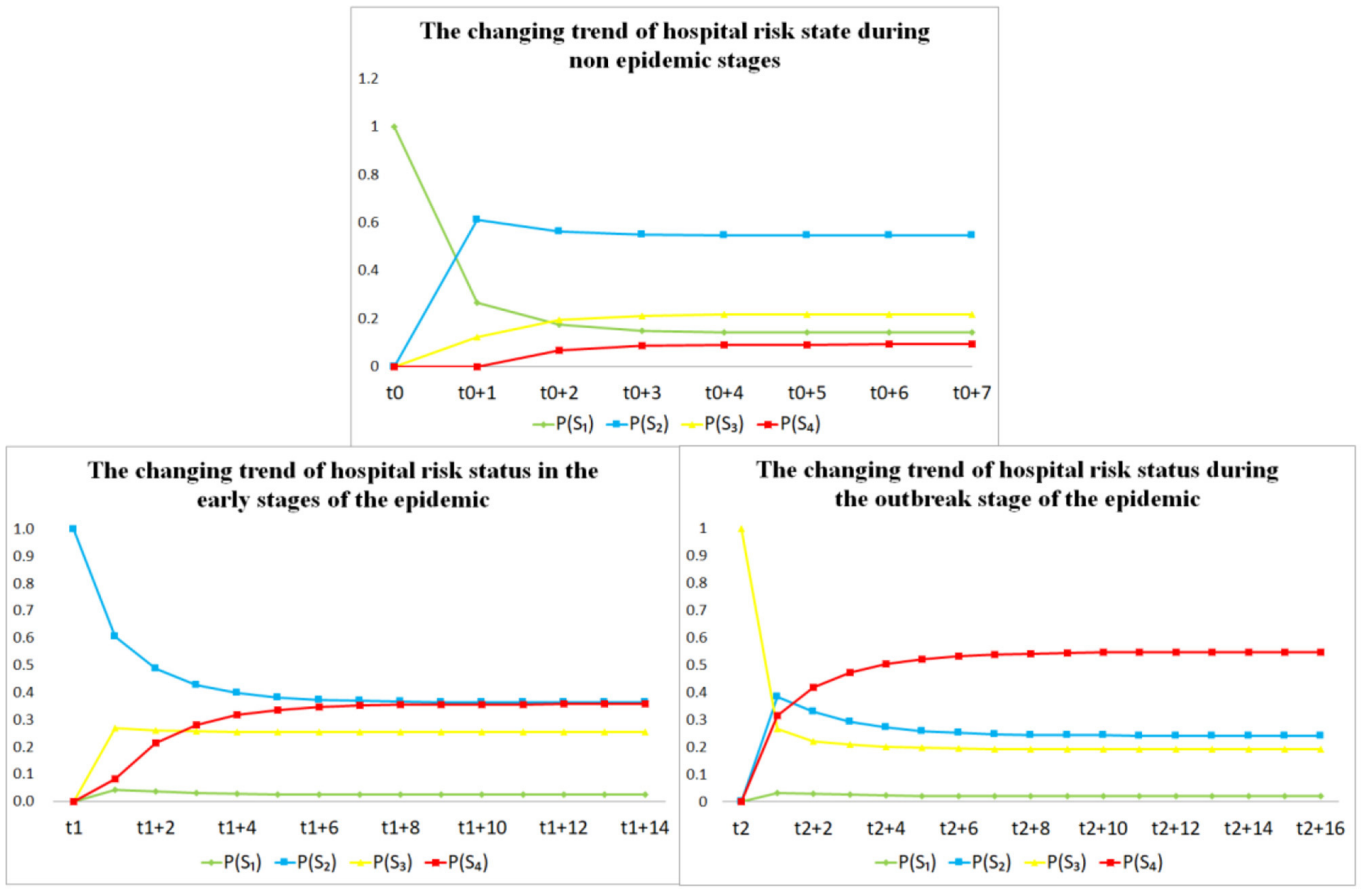
The epidemic risk state change trend in three different stages.

After calculating the risk state transition matrix *STM* of hospitals in different epidemic stages, according to Formula 3, it can be concluded that the final stable risk state of the epidemic only depends on *STM* and is independent of the risk state at time *t*. Therefore, it can be assumed that in the *t*0, *t*1, and *t*2 stages, the epidemic risk state of hospitals in the region is $P^{t0}\left(S_{n}\right)=\left\{1,0,0,0\right\},\;P^{t1}\left(S_{n}\right)=\left\{0,1,0,0\right\}$, and $P^{t2}\left(S_{n}\right)=\left\{0,0,1,0\right\}$. According to Formula 3, the epidemic stable risk states in three different stages can be calculated as ${\hat{P}}^{t0}\left(S_{n}\right)=\left\{0.142,0.546,0.220,0.093\right\}$, ${\hat{P}}^{t1}\left(S_{n}\right)=\left\{0.025,0.364,0.254,0.357\right\}$, and ${\hat{P}}^{t2}\left(S_{n}\right)=\left\{0.020,0.241,0.191,0.548\right\}$, respectively. The change trend of the epidemic risk state in these three stages is shown in Figure 3.

## 4 Discussion

### 4.1 Epidemic risk state and its change trend in different stages

It can be inferred from the assessment results of the risk state and its change trend in the three stages mentioned above.

(1) In stage *t*0, the value of ${\hat{P}}^{t0}\left(S_{2}\right)$ is significantly highest, indicating that there is a high probability that the epidemic risk state of hospitals in the region will transition toward *S*_2_ during non-epidemic stage. In the non-epidemic stage, these hospitals are in a relatively safe risk state, but there is already a relatively small epidemic risk in these hospitals.

(2) In stage *t*1, the high values of ${\hat{P}}^{t1}\left(S_{2}\right)$ and ${\hat{P}}^{t1}\left(S_{4}\right)$ indicate a high probability that these hospitals' risk state will transition toward *S*_2_ or *S*_4_ in the early epidemic stage. In the early epidemic stage, hospitals in the region faced significant risks and hidden dangers. The values of ${\hat{P}}^{t1}\left(S_{2}\right)$ and ${\hat{P}}^{t1}\left(S_{4}\right)$ are relatively close, indicating that these hospitals may transition toward the high-risk state of *S*_4_ if there is a slight mistake at this stage.

(3) In stage *t*2, the ${\hat{P}}^{t2}\left(S_{4}\right)$ value is significantly highest, indicating a high probability that these hospitals' risk state will transition toward *S*_4_ during the epidemic outbreak stage. During the epidemic outbreak stage, hospitals in the region are facing extremely serious risks, with a high probability of suffering serious losses. The values of ${\hat{P}}^{t0}\left(S_{1}\right)$ and ${\hat{P}}^{t0}\left(S_{2}\right)$ are both small, indicating that if hospitals in the region do not make timely adjustments at this stage, there is only a small probability that they can recover to a safe state.

As mentioned above, in the *t*0 stage, hospitals in the region are in a relatively safe state, but there is always a potential small risk of the epidemic. In stages *t*1 and *t*2, there is a high probability that the epidemic risk state will transition toward a more dangerous state.

### 4.2 Risk control priority analysis in different stages

Next, in order to assist the hospital in reasonable risk management in different stages, this paper intends to use the proposed assessment method to compare the effectiveness of different risk management plans, identify key risk indicators that affect these hospitals' risk state change, and provide priority reference for risk management.

In stages *t*1 and *t*2, considering that the impact of risk loss in the short term is not easily changed in the actual risk control process, hospitals can only reduce the risk occurrence probability to a certain extent. Therefore, in the assessment process of each indicator control plan, this paper intends to reduce the minimum occurrence probability level *P*_min_(*a*_*j*_) and the maximum occurrence probability level *P*_max_(*a*_*j*_) by one level, respectively, to simulate different risk indicator control plans.

Finally, the proposed method was used to assess the risk state after different controls, and the results are shown in Tables 8, 9.

**Table 8:** Assessment results of hospital risk state after only controlling *C*_*j*_ in stage *t*1.

** ${\text{P}}^{\text{t}1}\left({\text{S}}_{\text{n}}\right)$ **	**No control**	**Control only C_1_**	**Control only C_2_**	**Control only C_3_**	**Control only C_4_**	**Control only C_5_**	**Control only C_6_**	**Control only C_7_**	**Control only C_8_**	**Control only C_9_**
$P^{t1}\left(S_{1}\right)$	0.025	0.022	0.049	0.085	0.022	0.024	0.022	0.022	0.049	0.055
$P^{t1}\left(S_{2}\right)$	0.364	0.363	0.380	0.340	0.363	0.355	0.363	0.363	0.380	0.362
$P^{t1}\left(S_{3}\right)$	0.254	0.292	0.226	0.222	0.292	0.286	0.292	0.292	0.226	0.224
$P^{t1}\left(S_{4}\right)$	0.357	0.323	0.345	0.353	0.323	0.335	0.323	0.323	0.345	0.359
$P^{t1}\left(S_{1}\right)+P^{t1}\left(S_{2}\right)$	0.389	0.385	0.429	0.425	0.385	0.379	0.385	0.385	0.429	0.417

**Table 9:** Assessment results of hospital risk state after only controlling *C*_*j*_ in stage *t*2.

** ${\text{P}}^{\text{t}1}\left({\text{S}}_{\text{n}}\right)$ **	**No control**	**Control only C_1_**	**Control only C_2_**	**Control only C_3_**	**Control only C_4_**	**Control only C_5_**	**Control only C_6_**	**Control only C_7_**	**Control only C_8_**	**Control only C_9_**
$P^{t1}\left(S_{1}\right)$	0.025	0.022	0.049	0.085	0.022	0.024	0.022	0.022	0.049	0.055
$P^{t1}\left(S_{2}\right)$	0.364	0.363	0.380	0.340	0.363	0.355	0.363	0.363	0.380	0.362
$P^{t1}\left(S_{3}\right)$	0.254	0.292	0.226	0.222	0.292	0.286	0.292	0.292	0.226	0.224
$P^{t1}\left(S_{4}\right)$	0.357	0.323	0.345	0.353	0.323	0.335	0.323	0.323	0.345	0.359
$P^{t1}\left(S_{1}\right)+P^{t1}\left(S_{2}\right)$	0.389	0.385	0.429	0.425	0.385	0.379	0.385	0.385	0.429	0.417

**Figure 4:**
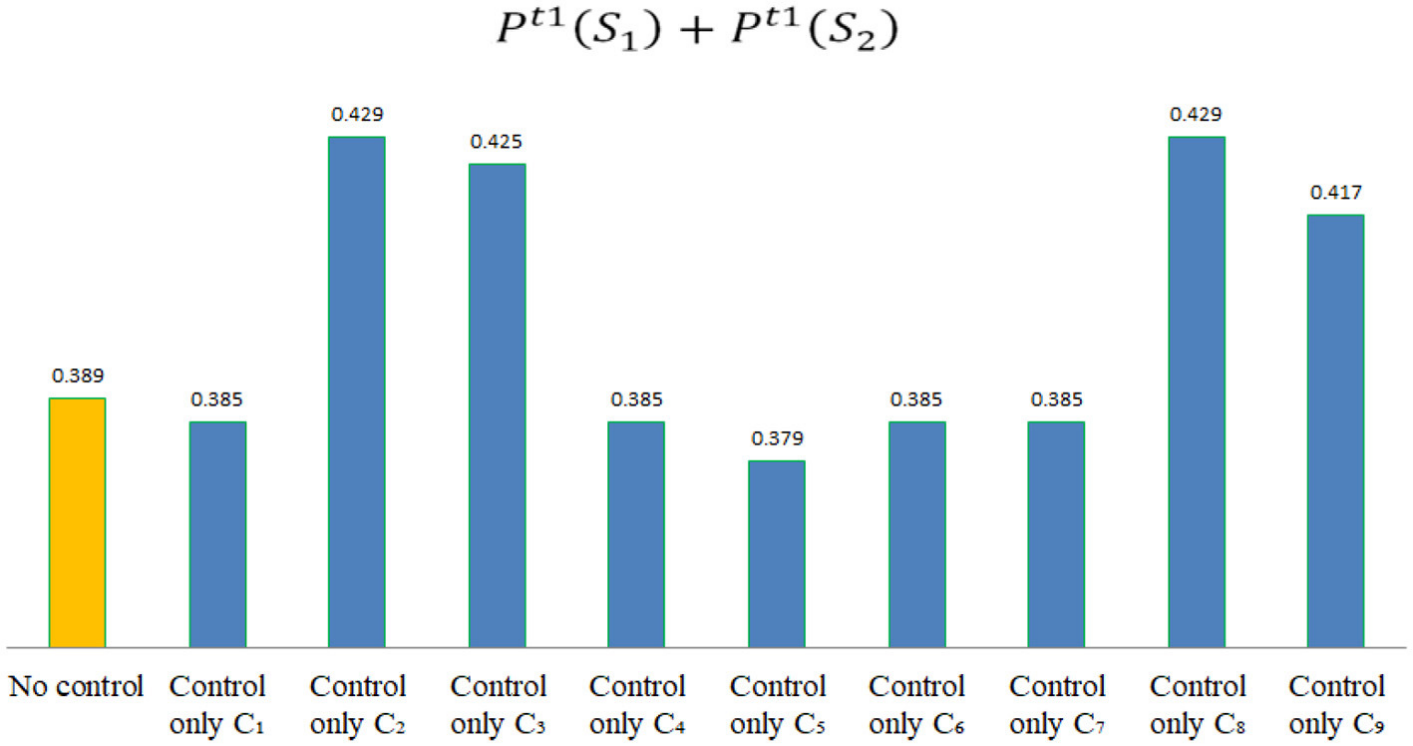
The value of $P^{t1}\left(S_{1}\right)+P^{t1}\left(S_{2}\right)$ after controlling different indicators in stage *t*1.

According to the assessment data shown in Table 8, if $P^{t1}\left(S_{1}\right)+P^{t1}\left(S_{2}\right)>0.389$, it indicates that the control of indicator *C*_*j*_ is effective in stage *t*1, which can enable the hospital to transition toward a safety risk state, as shown in Figure 4. By comparison, the following conclusions can be drawn.

(1) In the early epidemic stage, risk indicators that can improve these hospitals' risk state include: *C*_2_, *C*_3_, *C*_8_, and*C*_9_.

(2) In the early epidemic stage, risk control for *C*_1_, *C*_4_, *C*_5_, *C*_6_, or *C*_7_ will not effectively improve these hospitals' epidemic risk state.

(3) In the early epidemic stage, compared to other risk indicators, risk control for *C*_2_ and *C*_8_ is the most effective way to improve the hospital's epidemic risk state. This indicates that in the early epidemic stage, hospitals should prioritize the training of epidemic prevention skills for medical staff and the management of public health in hospitals. Secondly, it is the risk control for *C*_3_, namely the management of work rights, work responsibilities and performance assessment during the epidemic period. Finally, it is the risk control for *C*_9_, namely strengthening the epidemic supervision and management in hospitals.

**Figure 5:**
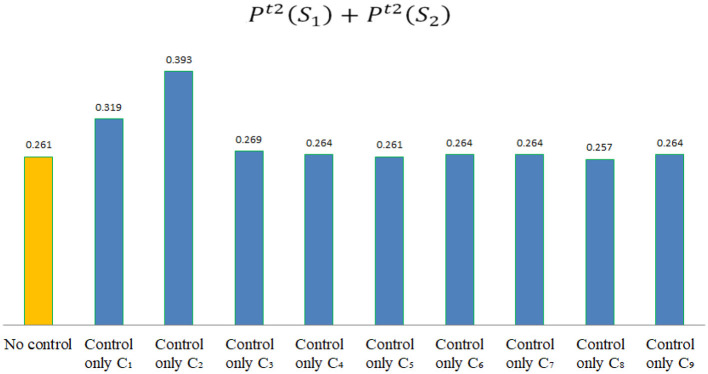
The value of $P^{t2}\left(S_{1}\right)+P^{t2}\left(S_{2}\right)$ after controlling different indicators in stage *t*2.

According to the assessment data shown in Table 9, if $P^{t2}\left(S_{1}\right)+P^{t2}\left(S_{2}\right)>0.261$, it indicates that the control of indicator *C*_*j*_ is effective in stage *t*2, which can enable these hospitals to transition toward a safety risk state, as shown in Figure 5. By comparison, the following conclusions can be drawn.

(1) In the epidemic outbreak stage, risk indicators that can improve these hospitals' risk state include: *C*_1_, *C*_2_, *C*_3_, *C*_4_, *C*_6_, *C*_7_, and *C*_9_.

(2) In the epidemic outbreak stage, risk control for *C*_5_ or *C*_8_ will not effectively improve these hospitals' epidemic risk state.

(3) In the epidemic outbreak stage, hospitals in the region first need to carry out risk control for *C*_2_ and *C*_1_, that is, prioritize training on epidemic prevention skills and psychological awareness for medical staff. Secondly, it is necessary to carry out risk control for the *C*_3_, namely strengthen the management of work rights, responsibilities, and performance evaluation during the epidemic period. Finally, it is also necessary to pay attention to the supply of emergency supplies in hospitals *C*_4_, the preparation of emergency personnel *C*_6_, and the management of public service facilities in hospitals *C*_7_.

The next step is to analyze the risk control plan for non-epidemic stage *t*0. In order to provide a priority reference for the risk control of hospitals in the region, this paper uses the same method to reduce the minimum probability level *P*_min_(*a*_*j*_) and maximum probability level *P*_max_(*a*_*j*_) of each risk indicator by one level. In this stage, if the *P*_min_(*a*_*j*_) of a certain risk indicator is already level 1, it will not decrease. Finally, by assessing the risk state after different controls, the results are shown in Table 10.

**Table 10:** Assessment results of hospital risk state after only controlling *C*_*j*_ in stage *t*0.

** ${\text{P}}^{\text{t}1}\left({\text{S}}_{\text{n}}\right)$ **	**No control**	**Control only C_1_**	**Control only C_2_**	**Control only C_3_**	**Control only C_4_**	**Control only C_5_**	**Control only C_6_**	**Control only C_7_**	**Control only C_8_**	**Control only C_9_**
$P^{t1}\left(S_{1}\right)$	0.025	0.022	0.049	0.085	0.022	0.024	0.022	0.022	0.049	0.055
$P^{t1}\left(S_{2}\right)$	0.364	0.363	0.380	0.340	0.363	0.355	0.363	0.363	0.380	0.362
$P^{t1}\left(S_{3}\right)$	0.254	0.292	0.226	0.222	0.292	0.286	0.292	0.292	0.226	0.224
$P^{t1}\left(S_{4}\right)$	0.357	0.323	0.345	0.353	0.323	0.335	0.323	0.323	0.345	0.359
$P^{t1}\left(S_{1}\right)+P^{t1}\left(S_{2}\right)$	0.389	0.385	0.429	0.425	0.385	0.379	0.385	0.385	0.429	0.417

**Figure 6:**
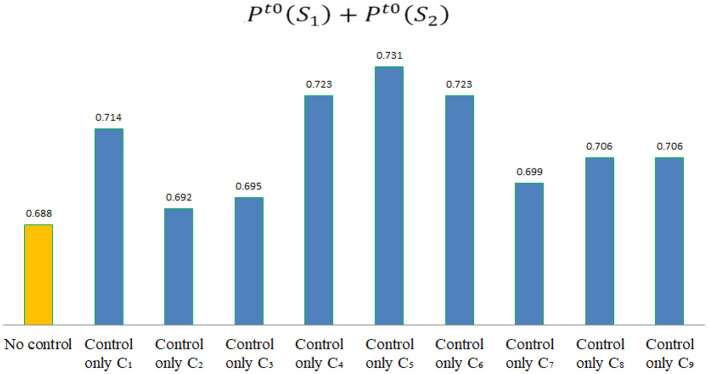
The value of $P^{t0}\left(S_{1}\right)+P^{t0}\left(S_{2}\right)$ after controlling different indicators in stage *t*0.

According to the assessment data shown in Table 10, if $P^{t0}\left(S_{1}\right)+P^{t0}\left(S_{2}\right)>0.688$, it indicates that the control of indicator *C*_*j*_ is effective in stage *t*0, which can enable these hospitals to transition toward a safety risk state, as shown in Figure 6. By comparison, the following conclusions can be drawn.

(1) In the non-epidemic stage, risk indicators that can improve these hospitals' risk state include: *C*_1_, *C*_2_, *C*_3_, *C*_4_, *C*_5_, *C*_6_, *C*_7_, *C*_8_, and*C*_9_.

(2) In the non-epidemic stage, risk control for *C*_5_ is the most effective, indicating that in the non-epidemic stage, the preparation of emergency funds in hospitals is the most important for ensuring hospital safety. In addition, training on the psychology and awareness of medical staff *C*_1_, the supply of emergency supplies *C*_4_, and the preparation of emergency personnel *C*_6_ are also important for ensuring hospital safety.

In summary, hospitals in underdeveloped areas should have different control strategies at different epidemic stages, that is, risk control for each indicator should have different priorities. By assessing the epidemic risk state and its change trend in different stages, scientific and reasonable risk control can be carried out (35). On the contrary, in the absence of effective assessment data, blind risk control may not be efficient and may even lead epidemic risk state to transition toward more dangerous state.

The method proposed in this paper takes into account the randomness and uncertainty of epidemic risk (36), viewing epidemic risk as a stochastic process under the influence of various risk indicators, and defining four random states of epidemic risk, achieving dynamic assessment of epidemic risk status and its change trend. Compared to general static risk assessment methods, this method establishes a random risk state space for the epidemic based on Markov theory, which is closer to the real epidemic risk environment. The assessment results can more effectively reflect the real epidemic risk situation in different periods, and the assessment results provided have higher support for decision-making.

## 5 Conclusion

This paper proposes the concept of epidemic risk state and proposes an effective assessment method for epidemic risk state and its change trend based on the risk matrix and Markov chain. Through assessment, reasonable priority references have been provided for risk management in underdeveloped area hospitals at different epidemic stages. For hospitals in underdeveloped areas, in the non-epidemic stage, hospitals should prioritize the preparation of emergency funds. In the early epidemic stage, hospitals should prioritize the training of epidemic prevention skills for medical staff and public health management in hospitals. During the epidemic outbreak stage, hospitals should prioritize training medical staff in epidemic prevention skills and psychological awareness. By using the method proposed in this paper to assess the epidemic risk state and its change trend in different stages, hospitals can effectively strengthen their ability to respond to sudden epidemics by implementing reasonable risk control measures in different stages. In subsequent research, in order to narrow the gap between the proposed method's assessment results and the actual epidemic risk state, it is necessary to further improve the epidemic risk indicator system established in this paper, establish a multi-level risk indicator system, and establish risk evidence corresponding to the risk occurrence probability and risk loss of each indicator. By assessing the risk level of each indicator through risk evidence, the accuracy and objectivity of the assessment can be improved.

## Data availability statement

The original contributions presented in the study are included in the article/Supplementary material, further inquiries can be directed to the corresponding author.

## Ethics statement

This study falls under the category of routine risk management policy evaluation in hospitals, does not require ethical approval or consent, all methods used in this paper were carried out in accordance with relevant guidelines and regulations, and all interview surveys in the study have obtained verbal authorization from all interviewees.

## Author contributions

MY: Funding acquisition, Writing – original draft, Writing – review & editing. JM: Conceptualization, Data curation, Writing – original draft. TL: Data curation, Methodology, Validation, Writing – review & editing. RJ: Formal analysis, Funding acquisition, Investigation, Methodology, Writing – review & editing. MJ: Data curation, Investigation, Writing – review & editing.
