# Supplementary material for: Assessment of epidemic risk state and its change trend of public hospital in underdeveloped area in different stages
Source: Front Public Health. 2024 Aug 6;12:1384118. doi: 10.3389/fpubh.2024.1384118 (PMC11333247; doi:10.3389/fpubh.2024.1384118)
Supplement: Supplementary file 1 [file Table_1.DOCX]

**The blank copy of the questionnaire**

**Q1: What is your current position in the hospital?**

- Doctors
- Nurses
- Administrative management personnel
- Logistics support personnel
- Other: [Please answer within this area]

**Q2: Have you participated in the psychological and epidemic prevention awareness training organized by the hospital for medical staff during the epidemic period?**

- Yes
- No

**Q3: Do you think it is necessary for hospitals to strengthen psychological and epidemic prevention awareness training for medical staff?**

- Very necessary
- Necessary
- General
- Not necessary
- Completely unnecessary

**Q4: How effective do you think the epidemic prevention awareness training organized by the hospital is?**

- Very satisfied
- Satisfied
- General
- Not satisfied
- Very dissatisfied

***Q5: Have you received training on epidemic prevention skills organized by hospitals for medical staff?**

- Yes
- No

**Q6: Do you think the content of epidemic prevention skills training is comprehensive and practical?**

- Very comprehensive and practical
- Comprehensive and practical
- General
- Not comprehensive or practical enough
- Very incomplete and impractical

**Q7: What areas do you think hospitals need to improve in the training of epidemic prevention skills for medical staff? (Multiple Choice)**

- The training content is not comprehensive enough
- The training methods are not flexible enough
- The training frequency is not high enough
- Insufficient training faculty
- Other: [Please answer within this area]

***Q8: Do you think hospital performance evaluation management was effective during the epidemic?**

- Very effective
- Effective
- General
- Invalid
- Completely ineffective

***Q9: Do you think the hospital has fully considered the work intensity during the epidemic period in performance evaluation management?**

- Very thoughtful
- Consider
- General
- Not very thoughtful
- Completely disregarding

***Q10: Do you think the hospital has sufficient emergency supplies?**

- Very abundant
- Adequate
- General
- Insufficient
- Very insufficient

***Q11: Do you understand the preparedness of emergency funds in hospitals?**

- Very knowledgeable
- Understanding
- General
- Not understanding
- Completely unaware

***Q12: Do you think there are any issues with emergency supplies in hospitals? (Multiple Choice)**

- Material shortage
- Uneven distribution of materials
- Delayed material updates
- Unstable material quality
- No problem

***Q13: Do you think the hospital is adequately prepared for emergency personnel?**

- Very sufficient
- Adequate
- General
- Insufficient
- Very insufficient

***Q14: How do you think hospitals should improve their emergency personnel preparedness? (Multiple Choice)**

- Increase the number of emergency personne
- Improve the training level of emergency personnel
- Improve the emergency personnel deployment mechanism
- No need for improvement
- Other: [Please answer within this area]

***Q15: Are you aware of the sources and usage of emergency funds in hospitals?**

- Very knowledgeable
- Understanding
- General
- Not understanding
- Completely unaware

***Q16: How do you think the management of hospital public service facilities (such as restrooms, rest areas, etc.) during the epidemic period?**

- Very satisfied
- Satisfied
- General
- Not satisfied
- Very dissatisfied

***Q17: Do you think the hospital is in place in terms of public health management (such as cleaning, disinfection, ventilation, etc.)?**

- Very well done
- In place
- General
- Not in place
- Very inadequate

***Q18: What areas do you think hospitals need to improve in terms of public service facility management?**

- Increase the number of facilities
- Improve facility cleanliness
- Strengthen facility maintenance
- No need for improvement
- Other: [Please answer within this area]

***Q19: Do you think the hospital's early warning and supervision management mechanism is effective during the epidemic?**

- Very effective
- Effective
- General
- Invalid
- Completely ineffective

***Q20: In what aspects do you think the hospital's early warning supervision and management mechanism needs to be strengthened?**

- The accuracy and timeliness of early warning information
- Transparency in monitoring and managing processes
- Response speed to emergencies
- No need for reinforcement
- Other: [Please answer within this area]

***Q21: What are your suggestions or opinions on the management of hospitals during the epidemic?**

- [Please answer within this area]

***Q22: Would you be willing to leave your contact information so that we can contact you in the future? (This information is not mandatory to fill in)**

- Phone number: [Please answer within this area]
